# Virtual Surgical Planning, Stereolitographic Models and CAD/CAM Titanium Mesh for Three-Dimensional Reconstruction of Fibula Flap with Iliac Crest Graft and Dental Implants

**DOI:** 10.3390/jcm10091922

**Published:** 2021-04-29

**Authors:** Carlos Navarro Cuéllar, Manuel Tousidonis Rial, Raúl Antúnez-Conde, Santiago Ochandiano Caicoya, Ignacio Navarro Cuéllar, Gema Arenas de Frutos, Ángela Sada Urmeneta, María Isabel García-Hidalgo Alonso, Carlos Navarro Vila, José Ignacio Salmerón Escobar

**Affiliations:** 1 Maxillofacial Surgery Department, Hospital General Universitario Gregorio Marañón, C/Doctor Esquerdo 46, 28007 Madrid, Spain; manuel@tousidonisrial.com (M.T.R.); antunezconde_92@hotmail.com (R.A.-C.); sochandiano@hotmail.com (S.O.C.); nnavcu@hotmail.com (I.N.C.); gema.arenas@gmail.com (G.A.d.F.); angela.sada15@gmail.com (Á.S.U.); hazamachado2@gmail.com (C.N.V.); jisalmeron@telefonica.net (J.I.S.E.); 2 Radiodiagnostic Department, Puerta de Hierro University Hospital, 28007 Madrid, Spain; mabelgha@gmail.com

**Keywords:** iliac crest graft, virtual surgical planning, CAD/CAM, titanium mesh, fibula flap

## Abstract

Mandibular reconstruction with fibula flap shows a 3D discrepancy between the fibula and the remnant mandible. Eight patients underwent three-dimensional reconstruction of the fibula flap with iliac crest graft and dental implants through virtual surgical planning (VSP), stereolitographic models (STL) and CAD/CAM titanium mesh. Vertical ridge augmentation and horizontal dimensions of the fibula, peri-implant bone resorption of the iliac crest graft, implant success rate and functional and aesthetic results were evaluated. Vertical reconstruction ranged from 13.4 mm to 10.1 mm, with an average of 12.22 mm. Iliac crest graft and titanium mesh were able to preserve the width of the fibula, which ranged from 8.9 mm to 11.7 mm, with an average of 10.1 mm. A total of 38 implants were placed in the new mandible, with an average of 4.75 ± 0.4 implants per patient and an osseointegration success rate of 94.7%. Two implants were lost during the osseointegration period (5.3%). Bone resorption was measured as peri-implant bone resorption at the mesial and distal level of each implant, with a variation between 0.5 mm and 2.4 mm, and with a mean of 1.43 mm. All patients were rehabilitated with a fixed implant prosthesis with good aesthetic and functional results.

## 1. Introduction

Mandibular defects derived from trauma, congenital malformations and tumor resection cause severe bone and soft tissue defects, with their consequent aesthetic and functional sequelae [1]. Segmental mandibulectomy leads to mandibular deviation, malocclusion, temporomandibular joint disorders and restriction to a soft diet [2]. Although the fibula flap has become the flap of choice for the reconstruction of composite mandibular defects, it does not provide sufficient height of bone to restore the native height of the mandible. The vertical discrepancy between the fibula flap and the native mandible results in (1) shortening of the vertical dimension of the lower third of the face with the consequent aesthetic defects and (2) difficulty in implant placement and prosthetic rehabilitation, which may cause implant overloading and endanger both the functional and aesthetic long-term results [3,4]. Therefore, vertical augmentation remains a challenge for oromandibular reconstruction. The main problem arises from the need to expand the soft tissue and achieve the proper bony architecture [5]. To solve this problem, iliac crest graft remains one of the main surgical options, as it supplies large quantities of cortico-cancellous bone. Bone grafting success depends on several factors: location of the grafting site, type of bone grafting materials, patient health, and physiologic conditions of the grafting site [6]. Physiologically, bone grafting success is a function of both neovascularization and graft integration [6]. The ultimate goal for bone grafting materials is the repair of bone to an extent that completely fills the defect and restores the former shape of the ridge [7]. The ingrowth of bone occurs through osteoconduction from the adjacent defect walls and follows the vascularization that invades the graft material. Therefore, the extent of bony regeneration is determined by the structure and size of the graft as well as the defect geometry [7]. The use of barrier membranes combined with autogenous bone grafting occlude the underlying defect from non-osseous tissue ingrowth, following the principles of guided bone regeneration. Without graft containment, soft tissues collapse, and graft displacement occurs. The titanium mesh used as a barrier produces consistent bone augmentation, maintains volume and regenerates high-quality bone for both vertical and horizontal mandibular reconstruction and dental implants. It provides superior space maintenance with predictable and consistent results. Furthermore, the pores within the titanium mesh are thought to play a critical role in maintaining blood supply to a grafted defect [8]. The main problem to achieve a correct three-dimensional reconstruction of the bone is the fitting of the titanium mesh over the peroneal flap and the complete filling of the mesh with the iliac crest graft. In standard techniques, this is difficult to achieve, resulting in mesh exposure, infection, graft displacement and, consequently, bone resorption [9].

The purpose of this study was to evaluate the outcomes of the three-dimensional reconstruction of the fibula flap with iliac crest graft and dental implants through virtual surgical planning (VSP), STL models and CAD/CAM titanium mesh. The specific aims of this study were to evaluate (1) the vertical ridge augmentation of the fibula flap; (2) the horizontal dimension of the fibula flap; (3) the peri-implant bone resorption of the iliac crest graft; (4) the implant success rate; (5) the functional results; and (6) the aesthetic results.

## 2. Materials and Methods

To address the research purpose, the investigators designed and implemented a retrospective study during a 5-year period (2014–2018) with 8 patients (5 men and 3 women) with segmental mandibular defects reconstructed with a fibula free flap at Hospital General Universitario Gregorio Marañón (Madrid, Spain). Three oncologic patients were diagnosed with squamous cell carcinoma, and three patients were diagnosed with ameloblastoma. Two patients with mandibular segmental defects resulting from traumatic injuries underwent secondary reconstruction. Six months later, vertical reconstruction of the fibula was performed in seven nonirradiated patients with an onlay iliac crest bone graft, while one patient received radiotherapy, and the graft was performed 1 year after the end of radiotherapy. Virtual surgical planning (VSP), stereolitographic models (STL) and a custom-made titanium mesh (CAD/CAM) (Maffinter^®^, Madrid, Spain) were designed prior to surgery to enable both vertical and horizontal reconstruction of the fibula flap. A two-team approach was accomplished and a cortico-cancellous graft from the anterior superior iliac crest was harvested due to its thicker cortical layer. As it was a cortico-cancellous graft and not a microsurgical flap, no cutting guides were used, and the adaptation of the graft to the CAD/CAM titanium mesh was performed using a standard freehand procedure. A cervical approach was performed to expose the fibula flap and remove the osteosynthesis material. In this way, the cortico-cancellous graft was isolated from the oral cavity. In all patients cortico-cancellous graft was fixed using CAD/CAM titanium mesh, adapted and fixed to the remaining fibula. Six months later, the three dimensions of the mandible were evaluated by the Radiology Department through CT Scan, providing relevant quantitative data regarding bone volume and bone resorption. The height of the graft was measured in both sagittal and coronal CT sequences, while the width of the graft was measured in the axial CT sequences. An intraoral approach was planned, the titanium mesh was removed and dental implants (Ticare^®^, Valladolid, Spain) were placed in all patients, who were subsequently rehabilitated with a fixed implant-supported prosthesis with the aim of achieving a comprehensive reconstruction, both aesthetic and functional.

In the postoperative follow-up, CT scan and CBCT were performed and evaluated by the Radiology Department of the Hospital to assess the increase in mandibular height and width achieved, as well as the bone resorption and the implant success rate. Aesthetic results were evaluated by the patients and morphing CT Scan. Functional results were evaluated 1 year after prosthetic rehabilitation. The criteria for implant success were immobility, absence of peri-implant radiolucency, adequate width of the attached gingiva and absence of infection. The study and review of the medical records and data collection and the subsequent analysis of the data collected are endorsed by the Hospital Ethics Committee. The inclusion criteria were: (1) Oncologic patients treated with segmental mandibulectomy and reconstructed with fibula flap; (2) Patients with traumatic injuries and mandibular segmental defects reconstructed with fibula flap; (3) Patients with three-dimensional discrepancy between the native mandible and the fibula flap; (4) VSP, stereolitographic models and CAD/CAM titanium mesh for 3D fibula reconstruction; and (5) Cortico-cancellous iliac crest graft. The exclusion criteria were (1) segmental mandibular defects reconstructed with double-barrel fibula flap and (2) Patients who previously underwent vertical distraction of the fibula flap. The variables evaluated in this study were:

1. Vertical augmentation of fibula flap. CT scan was performed in the postoperative follow-up, and vertical augmentation was defined as that adding to the height of the existing bone.
2. Horizontal dimensions of fibula flap. The horizontal dimension of the fibula flap was studied to verify that the increase in height was able to maintain the previous horizontal dimension of the fibula.
3. Bone resorption. CT scan and CBCT were performed to assess the peri-implant bone resorption 3 years after prosthetic rehabilitation of dental implants.
4. Implant success rate was evaluated 3 years after prosthetic rehabilitation.
5. Aesthetic results. Esthetic assessment by the patients was performed to address scores in facial symmetry, facial scaring and facial projection. The results were classified with scores 0 (“poor”), 1 (“fair”) and 2 (good”).
6. Functional results. All patients were rehabilitated with Ticare^®^ implants (Valladolid, Spain) and fixed implant-supported prosthesis. Speech articulation and deglutition were evaluated. Deglutition was assessed and the results were classified with scores 0 (liquid diet), 1 (soft diet) and 2 (regular diet). Speech articulation was evaluated as intelligible language and unintelligible language.

## 3. Results

Eight patients with segmental mandibular defects were reconstructed with a free fibula flap. Three-dimensional reconstruction was performed through VSP, cortico-cancellous iliac crest graft and CAD/CAM titanium mesh. The follow-up period was from 2 years 4 months to 4 years 11 months (average 3 years 8 months). In six patients, the origin was oncological, 3 squamous cell carcinoma and 3 ameloblastomas. Two patients were secondarily reconstructed due to traumatic sequelae. The patients had an average age of 47.75 years. Five patients were men (62.5%) and three patients were women (37.5%). The smallest mandibular segmental defect was 8.9 cm, and the largest defect was 12.6 cm, with an average of 10.05 cm. (Table 1). Seven patients did not receive radiation therapy (87.5%), and the vertical reconstructive surgery was performed 6 months later, while one patient was irradiated (12.5%) and the second surgery was deferred 12 months after radiotherapy. The original height of the fibula flap ranged from 12.1 mm to 14.4 mm, with an average of 13.55 mm. The transverse dimension of the fibula was measured in the VSP. Iliac crest graft and titanium mesh were able to preserve the width of the fibula, which ranged from 8.9 mm to 11.7 mm, with an average of 10.1 mm. Reconstruction of fibula height was measured by the Radiology Department. Each segment of the iliac crest graft was evaluated by measuring the vertical bone gain from the top of the fibula to the top of the iliac crest graft. Three points were measured in each segment (medial, central and lateral) at the points of highest bone gain. Vertical reconstruction ranged from 13.4 mm to 10.1 mm, with an average of 12.22 mm. (Table 1).

The vertical reconstruction of the fibula with the iliac crest graft doubled the original height of the fibula flap. No titanium mesh exposure was observed, and none of the grafts communicated with the oral cavity. Six months later, once the ossification of the graft was checked by panoramic radiograph and CT scan, an intraoral approach was accomplished, the titanium meshes were removed and dental implants were placed (Ticare^®^, Valladolid, Spain). A total of 38 implants were placed in the new mandible, with an average of 4.75 ± 0.4 implants per patient and an osseointegration success rate of 94.7%. Two implants were lost during the osseointegration period (5.3%). All patients were rehabilitated with a fixed implant prosthesis. Bone resorption was measured as peri-implant bone resorption, 3 years after prosthetic rehabilitation, through CBCT. The measurement was performed at the mesial and distal level of each implant, with a variation between 0.5 mm and 2.4 mm, and with a mean of 1.43 mm. Six patients reported a good aesthetic result while two patients described the result as fair. The morphing images showed a significant improvement in the lower facial third between the initial mandibular reconstruction and the final results after iliac crest graft, implant placement and prosthetic rehabilitation. In terms of functional results, speech articulation was evaluated as intelligible language in all patients. Seven patients reported a regular diet while one patient reported a soft diet.

### Case Presentation

**Figure 1:**
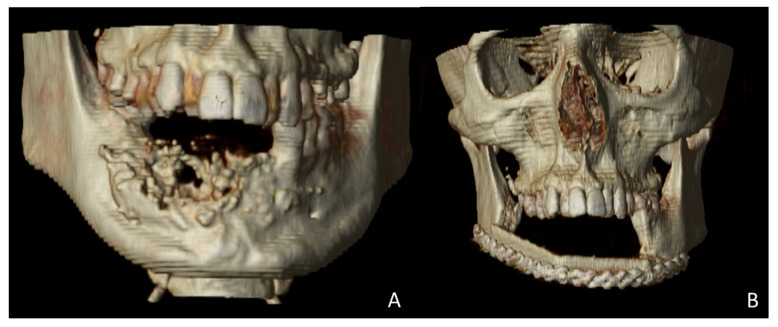
Postoperative 3D scan. (**A**) CT Scan showing destruction of the mandibular symphysis due to ameloblastoma. (**B**) CT Scan with segmental mandibulectomy from left mandibular body to right mandibular angle and immediate reconstruction with fibula free flap.

A 38-year-old patient was referred to our Department reporting progressive deformity of the mandibular symphysis with loss of teeth. Panoramic radiography and 3D CT scan showed a lytic lesion with destruction of the external mandibular cortex (Figure 1A).

**Figure 2:**
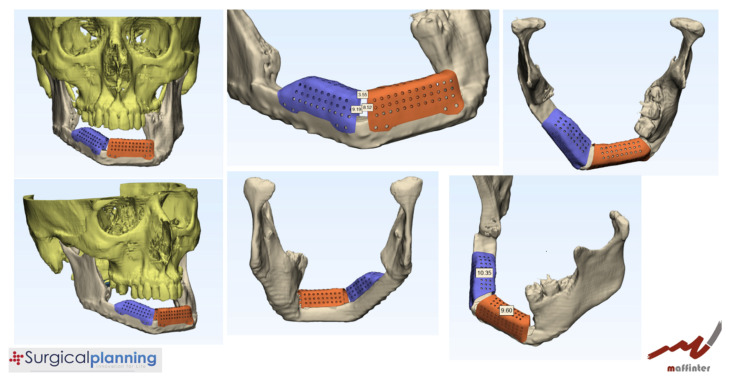
VSP for vertical reconstruction of the fibula with two CAD/CAM titanium meshes adapted to the two segments of the fibula flap.

The patient was diagnosed with a mandibular ameloblastoma, affecting the mandibular symphysis and both mandibular bodies. Tumor resection with segmental mandibulectomy and clear margins, and immediate reconstruction with a two-segment fibula flap was performed (Figure 1B). Six months later, a vertical discrepancy between the remnant mandible and the fibula flap was assessed and a virtual surgical planning (VSP) with a cortico-cancellous iliac crest graft was planned. VSP was performed with the biomedical engineer (Maffinter^®^, Madrid, Spain) and a three-dimensional virtual reconstruction of the defect was performed with two titanium CAD/CAM meshes (Figure 2) with 1.5 mm diameter pores.

**Figure 3:**
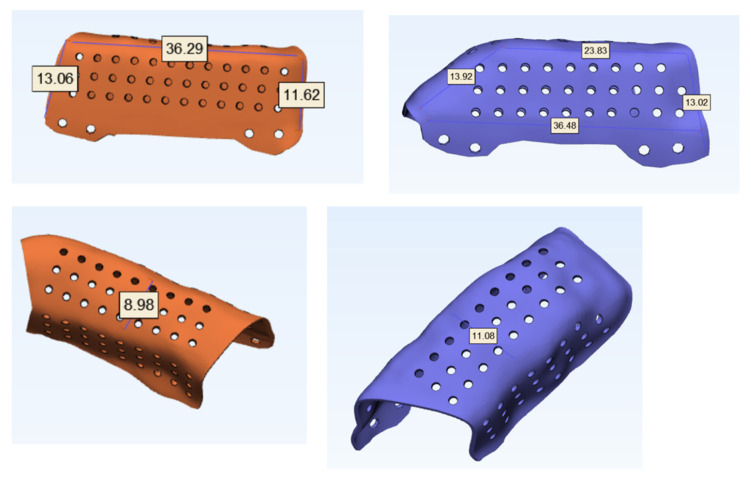
Vertical and horizontal dimensions of titanium customized meshes (CAD/CAM). Mesh porosity to allow for revascularization and bone regeneration.

**Figure 4:**
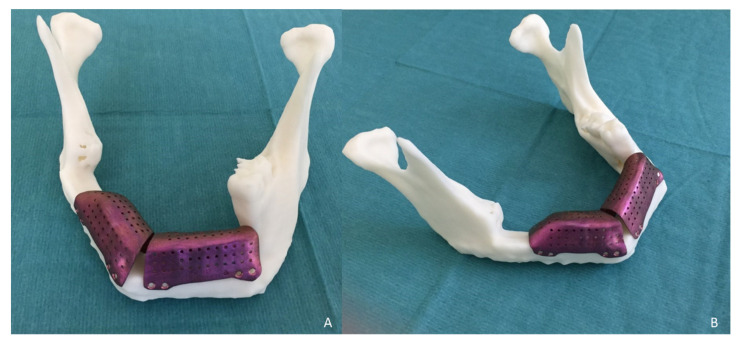
(**A**) and (**B**) Stereolithographic model (STL) showing the vertical bone discrepancy and the adaptation of CAD/CAM titanium mesh to the fibula for three-dimensional bone reconstruction.

**Figure 5:**
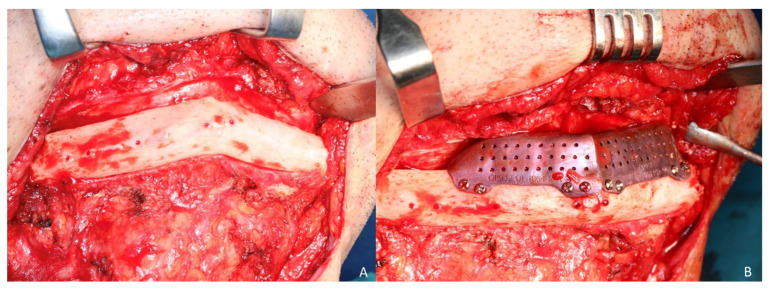
3D reconstruction of fibula flap. (**A**) Cervical approach to avoid intraoral communication and exposure of the peroneal flap with good bone vascularization. (**B**) Iliac crest cortico-cancellous graft to reconstruct mandibular height and preserve the horizontal dimension of the fibula. Adjustment of the titanium mesh to the upper and lateral part of the two fibula segments with 1.5 mm screws.

The measurements of the meshes were 36 × 13 × 9 mm and 23 × 13 × 11 mm (Figure 3). STL model and CAD/CAM titanium mesh were printed and checked before surgery (Figure 4). Under general anesthesia, a cervical approach was performed to expose the fibula and remove the osteosynthesis material without communicating with the oral cavity (Figure 5A).

**Figure 6:**
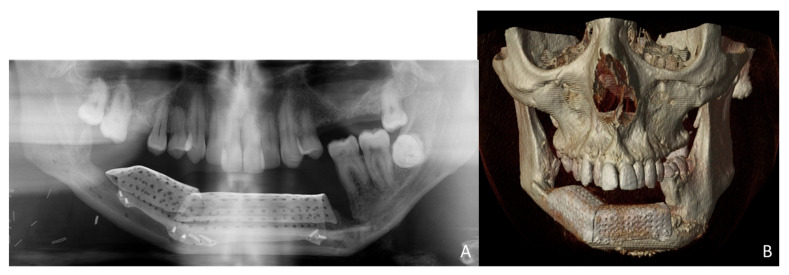
Postoperative images after 3D reconstruction with CAD/CAM titanium mesh and iliac crest graft. (**A**) Panoramic radiograph showing the vertical reconstruction of the symphysis and both mandibular bodies with the iliac crest graft. (**B**) CT Scan with three-dimensional reconstruction of the mandible.

**Figure 7:**
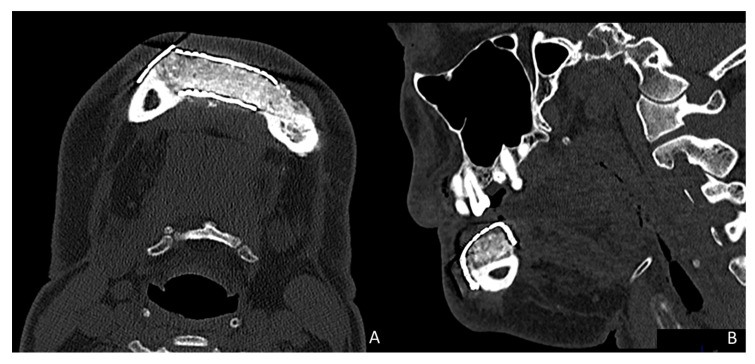
Postoperative CT scan. (**A**) Axial CT Scan demonstrating the stability of the transverse dimension of the fibula with respect to the remnant mandible. (**B**) The three-dimensional preservation of the iliac crest graft with CAD/CAM mesh makes it possible to double the height of the fibula.

Simultaneously, a cortico-cancellous graft of the left anterosuperior iliac crest was obtained. The graft was fixed to the fibula using the CAD/CAM titanium mesh and 1.5 mm screws (Figure 5B). There was no intraoral exposure of the graft and an increase in the vertical dimension of the fibula was achieved and demonstrated by panoramic radiograph and CT scan (Figure 6A,B and Figure 7A,B).

**Figure 8:**
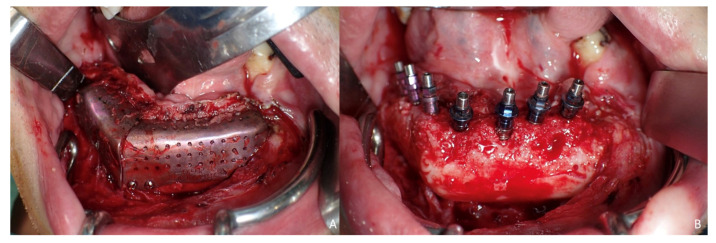
Ossification of the graft. (**A**) Intraoral approach showing the three-dimensional preservation of the customized mesh. (**B**) Removal of the titanium mesh. Accuracy of vertical reconstruction of the fibula with preservation of the horizontal dimension of the bone and immediate placement of Ticare^®^ dental implants.

**Figure 9:**
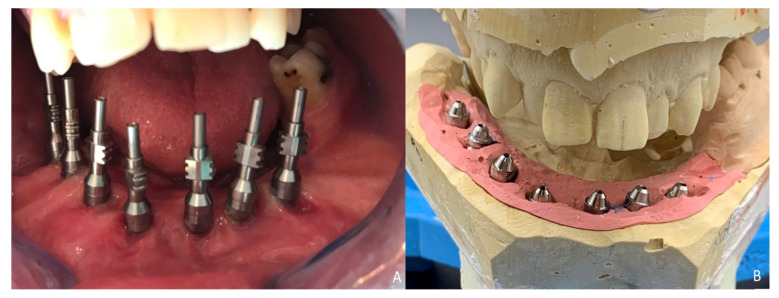
Prosthetic rehabilitation. (**A**) Second-stage surgical procedure and impression copings for dental rehabilitation with successful soft-tissue reconstruction. (**B**) Evaluation of the occlusal vertical dimension and record centric relation.

**Figure 10:**
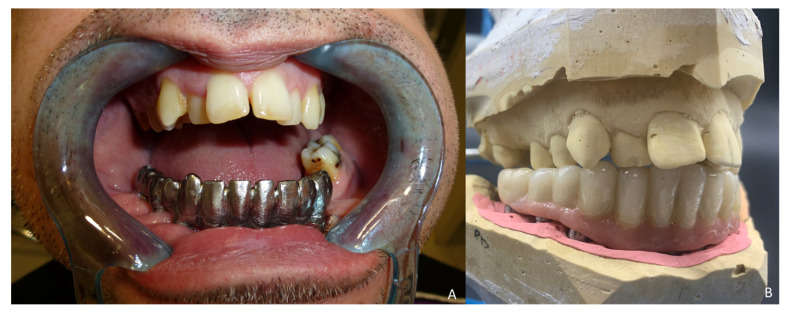
Implant prosthesis. (**A**) Metal framework evaluated intraorally. (**B**) Functional rehabilitation with a ceramic fixed implant-supported prosthesis.

Six months later, the ossification of the graft and volume of the soft tissue were verified. An intraoral approach was performed, the titanium mesh was removed and the increase in height and width achieved with the graft was verified, showing the space beneath the titanium mesh completely filled with new hard tissue (Figure 8A). Seven dental implants were placed (Ticare^®^, Valladolid, Spain) (Figure 8B), and, four months later, the second surgical procedure of the implants was performed (Figure 9A). Evaluation of the occlusal vertical dimension and record centric relation was accomplished (Figure 9B) and the metal framework was evaluated intraorally (Figure 10A).

**Figure 11:**
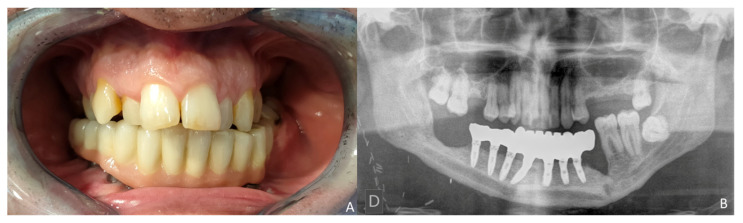
Functional result. (**A**) Final dental restoration. (**B**) Panoramic radiograph demonstrating the reconstruction of the previous height of the mandible with a correct osseointegration of the implants.

**Figure 12:**
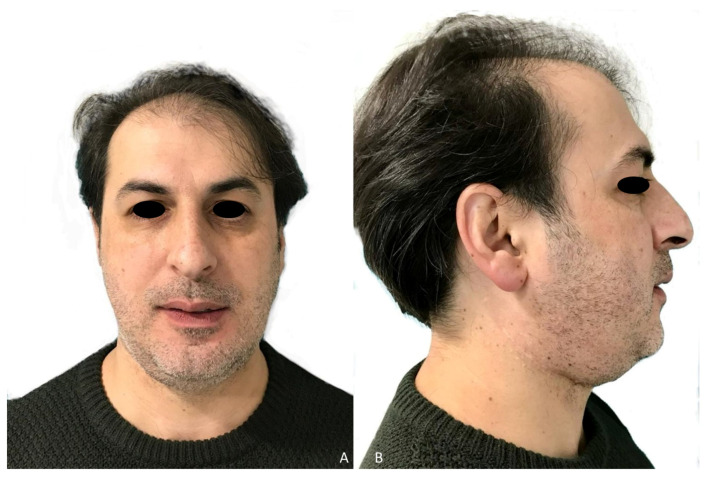
Final aesthetic result. (**A**) Aesthetic result with mandibular symmetry. (**B**) Aesthetic profile with a good projection of the lower facial third.

The prosthetic rehabilitation was carried out by means of a fixed implant-supported prosthesis providing normal occlusion (Figure 10B and Figure 11A). Three years later, there was no evidence of significant peri-implant bone resorption (Figure 11B). The prosthetic rehabilitation allowed a correct aesthetic and functional result with a regular diet and intelligible speech (Figure 12A,B).

**Figure 13:**
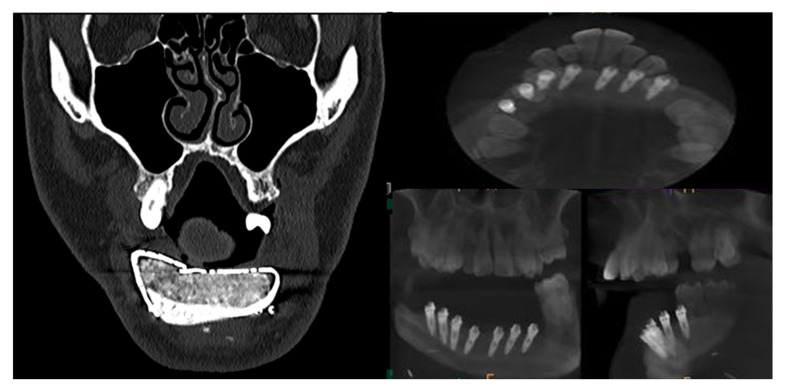
CT Scan and CBCT were performed in the postoperative follow-up, providing relevant quantitative data regarding bone volume and bone resorption.

**Figure 14:**
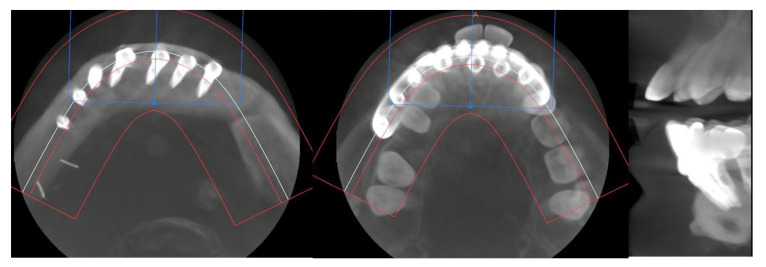
CBCT performed in the follow-up to evaluate bone resorption before and after implant loading.

**Figure 15:**
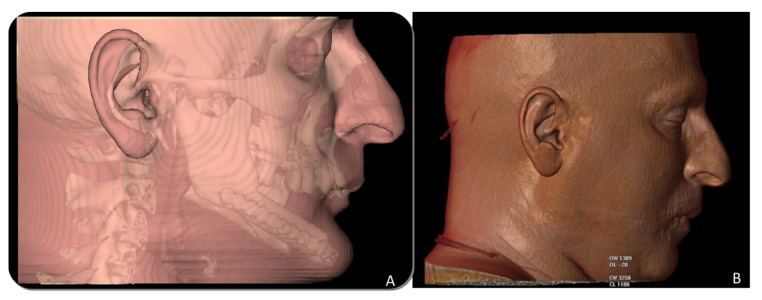
Aesthetic comparison. (**A**) Morphing performed through CT Scan after mandibular reconstruction with fibula free flap, showing a retrusion and collapse of the lower facial third. (**B**) Morphing performed after implant rehabilitation, demonstrating a significant aesthetic improvement after 3D reconstruction with iliac crest graft.

CT Scan and CBCT were performed in the postoperative follow-up, and bone volume and bone resorption (Figure 13 and Figure 14) were evaluated by the Radiology Department of the hospital. A morphing reconstruction was performed to compare the lower facial third showing an improvement in the aesthetic profile and facial projection (Figure 15).

## 4. Discussion

Mandibular segmental defects lead to significant aesthetic and functional sequelae, and primary reconstruction is mandatory. Although the fibula free flap is the most versatile technique for reconstruction, its main disadvantage is the low height of bone to reconstruct the height of the previous mandible [2,4]. In cases of mandibular segmental defects with a remaining mandible and occlusion on one or both sides of the defect, there is a vertical discrepancy between the fibula and the remnant mandible, which makes the correct prosthetic rehabilitation with implants impossible because the prosthesis would be extremely high and occlusally inadvisable [4]. The purpose of this study was to evaluate the outcomes of the three-dimensional reconstruction of the fibula flap with iliac crest graft and dental implants through virtual surgical planning (VSP) and CAD/CAM titanium mesh.

Although the sample size of this study was small, it is the first study that demonstrates the efficacy and stability of 3D reconstruction of fibula flaps with iliac crest graft through VSP and CAD/CAM titanium mesh and the oral rehabilitation with dental implants and fixed implant-supported prosthesis. Different studies have previously reported the vertical reconstruction of the mandible with different grafts, but none of them have reported on a peroneal flap through VSP and CAD/CAM titanium mesh. Verhoeven [10] reported a two-dimensional study, showing a bone resorption of approximately 25% of the height of the graft. Vermeeren [11] described a resorption of grafted iliac bone ranging from 44 to 50%. Johansson [12] evaluated the volume of iliac crest grafted at 6-month post-operative controls with an average volume reduction of 47 to 50%, while Smolka [13] reported a 19% resorption for calvarial grafts at 1-year follow-up. Hertford [6] evaluated the use of titanium mesh grafting combined with rhBMP-2 for alveolar reconstruction, with optimal results, and Casap [14] reported the use of printed titanium shell with BMP-2/allograft for vertical alveolar augmentation, showing the bone fill of 90% but with a high rate of mesh exposure. In this study the investigators demonstrate that 3D reconstruction of the fibula can be reliably performed using an iliac crest graft and a titanium mesh planned through VSP and CAD/CAM. The customized mesh adapts optimally to the fibula and the surrounding soft tissues, offering three-dimensional support. Likewise, graft stability is observed at 3 years postoperatively, with minimal bone resorption measured with follow-up CBCT and evaluation by the Radiology Department. The correct osseointegration of the implants was observed, enabling a reliable occlusal rehabilitation in the long term and an optimal aesthetic and functional result.

The goal of tissue engineering is to regenerate missing tissues by combining cells from the body and highly porous scaffolds that act as templates for tissue regeneration and guide the growth of new tissues [15]. Therefore, three elements are needed for tissue regeneration: a source of cells, signaling molecules and a matrix [16]. As for bone regeneration, autogenous grafts, such as the iliac crest graft, remain the gold standard, given that they have osteogenic potential in addition to their osteoinductive and osteoconductive properties. The success of bone grafting depends on the 3D features of the bone graft (shape, external surface, thickness and height), the volume of the recipient site and the relationship between the two. Bone remodeling depends on the recipient site and the graft sources. For vertical reconstruction, the fibula provides an optimal recipient site with good vascularity and sufficient bone width for the positioning of the graft with the titanium mesh. The iliac crest graft for vertical reconstruction offers several advantages [17]: (1) sufficient cellular quality; (2) sufficient quantity of graft material; (3) compacting cells to increase density; (4) rich vascularization; and (5) reliability and stability in recipient sites with good vascularity. On the other hand, it has several disadvantages: (1) requiring a second surgical procedure; (2) the morbidity derived from the approach to the iliac crest; (3) the possibility of exposure of the titanium mesh and bone graft, especially in irradiated patients; and (4) the need to wait 6 months for ossification before placing the implants, making the prosthetic rehabilitation longer. This technique is indicated in patients who are not going to receive radiotherapy and in patients with extensive defects at the level of the symphysis and mandibular body. When this technique is accomplished, it is important to perform a cervical approach for the placement of the graft and avoid communication with the oral cavity [4]. In this way, we avoid mesh exposure, graft infection and bone resorption.

One point of discussion would be whether it is better to perform a double-barrel fibula flap, as the 3D reconstruction is primarily performed, and no delayed surgery is needed compared with the vertical distraction or cortico-cancellous iliac crest grafts that require multiple stages to reach the final outcomes [4]. The double-barrel fibula flap is the ideal technique to reconstruct the mandibular height and to solve the problem of vertical discrepancy. Furthermore, virtual surgical planning (VSP), computer-aided design/computer-aided manufacturing (CAD/CAM), surgical navigation and advanced implantology allow three-dimensional reconstruction of the original jaw, fewer surgical stages and faster prosthetic rehabilitation with high accuracy [4]. The disadvantage of the double-barrel technique is that it is not advisable to reconstruct defects greater than 10 cm, because the length required is almost 24 cm, which can lead to higher donor site morbidity and, therefore, is not always possible to perform.

One of the most important factors to achieve an optimal aesthetic and functional result is to use a mesh that allows us to maintain the three-dimensional reconstruction of the bone. Stable space maintenance is an essential component of all bone development, repair and regeneration [14]. Titanium mesh has been shown to be ideal for this procedure due to its characteristics and benefits: (1) it is a biocompatible material; (2) it maintains space and prevents collapse of the graft due to its rigidity; (3) it is non-resorbable, and prevents the soft tissue invasion into the grafting material; (4) it provides a smooth surface to prevent bacterial colonization; (5) its elasticity prevents compression of the intraoral soft tissue; (6) small pores maintain the blood supply to the grafted defect; and (7) it allows intraoperative modifications. The main disadvantages are the cost and the need of a second surgery to remove the mesh and place the dental implants, as well as the possibility of mesh exposure. Although several studies have addressed that titanium mesh exposure leads to early graft resorption of about 15% to 25% [18,19], our study reveals the absence of titanium mesh exposure and minimal bone resorption. These results are due to: (1) the cervical approach that allows us not to communicate with intraoral tissues, avoiding graft contamination; (2) the VSP allows us to perform a precise and accurate three-dimensional reconstruction of the defect to be reconstructed, being able to design the dimensions of the mesh and avoiding tension in the surgical wound and compression of the gum on the titanium mesh; and (3) CAD/CAM allows us to design and print the titanium mesh with smooth surface and angles and pores < 2 mm, with the consequent fitting to the fibula flap and the gum, allowing the vascularization of the graft.

The precise fitting and shaping of the titanium mesh with the iliac crest bone graft to the fibula flap is critical in order to achieve optimal functional and aesthetic outcomes. Standard surgery may cause the mesh to form an inappropriate shape, leading to wound dehiscence, bone resorption, dead spaces and infection [9]. A virtual surgical planning with stereolithographic models and CAD/CAM titanium mesh enables the recreation and maintenance of the mandibular shape, promotes proper alignment of the graft for implant placement and dental occlusion, achieves a correct orthognathic relationship, increases reconstruction accuracy and decreases surgical time [17]. One potential limitation of the VSP is the added cost. Nevertheless, the improved patient outcomes demonstrated with VSP need to be balanced against the added cost of the technology. Future studies are needed to evaluate the accuracy achieved compared with standard surgery and the total value added and the cost efficiency of VSP-CAD/CAM. Overall, the decreased patient morbidity and complications and the generalized improved outcomes may potentially offset the technological costs.

From the esthetic point of view, it is advisable to reconstruct the original dimensions of the mandible to achieve a good projection of the lower facial third. Otherwise, and despite the prosthetic rehabilitation of the implants, this three-dimensional bone defect, if not reconstructed, can cause the long-term collapse of the soft tissues at the level of the chin, with consequent aesthetic sequelae, especially if the previous vertical dimension of the mandible has not been reconstructed.

Finally, it is important to emphasize that mandibular segmental defects reconstructed with the fibula flap can be reconstructed three-dimensionally with cortico-cancellous iliac crest grafts through VSP, STL models and CAD/CAM titanium mesh. These techniques allow for an accurate 3D reconstruction of the mandible with minimal bone resorption and long-term stability. The combination of these surgical techniques together with advanced implantology allows the comprehensive rehabilitation of these patients, providing aesthetic and functional results that return quality of life to patients.

## 5. Conclusions

The multi-stage implementation of virtual surgical planning (VSP) with the use of stereolithographic models (STL) and CAD/CAM titanium mesh for 3D mandibular defects offers a reconstructive accuracy, which was previously reliant on surgeon experience and intraoperative trial-and-error using 2D imaging modalities. It increases bone-to-bone contact, has a better mesh alignment and improves operative efficiency with reduced complication rates and minimal bone resorption. It provides an accurate three-dimensional reconstruction of the fibula flap, which leads to an optimized implant placement and dental alignment, thereby enhancing facial symmetry, aesthetic contour and function.

## Figures and Tables

**Table 1:** Fibula reconstruction with iliac crest bone graft through VSP and CAD/CAM titanium mesh.

Gender/Age (Years)	Diagnosis	Length of Defect (cm)	Vertical Reconstruction (mm)	Vertical Fibula Height (mm)	Horizontal Dimensions (mm)	Number of Implants/Failure	Boneresorption (mm)	Radiotherapy	Aesthetic Result	Functional Result
M/13	Traumatic injury	10.5	12.7	13.6	10.1	5	1.4	No	2	2
M/42	Ameloblastoma	9.5	11.7	14.1	11.2	4	1.5	No	2	2
F/61	Squamous cell carcinoma	10.8	11.9	12.3	9.6	4	1.5	No	2	2
F/63	Squamous cell carcinoma	9.6	12.3	12.1	9.4	4	1.6	No	1	2
M/57	Traumatic injury	9.1	12.5	13.8	10.4	6 (1 failure)	1.2	No	2	2
M/38	Ameloblastoma	12.6	13.2	14.4	11.7	7	0.5	No	2	2
F/35	Ameloblastoma	9.4	13.4	13.9	9.7	4	1.4	No	2	2
M/73	Squamous cell carcinoma	8.9	10.1	14.2	8.9	4 (1 failure)	2.4	60 Gy	1	1
Average		10.05 cm	12.22 mm	13.55 mm	10.1 mm	38 (2 failures): 94.7%	1.43 mm			

## Data Availability

The data presented in this study are available on request from the corresponding author. The data are not publicly available due to data protection regulations.
